# Astaxanthin and DHA supplementation ameliorates the proteomic profile of perinatal undernutrition-induced adipose tissue dysfunction in adult life

**DOI:** 10.1038/s41598-023-38506-x

**Published:** 2023-07-29

**Authors:** Anu V. Ranade, Pramukh Subrahmanya Hegde, Megha Agni Bhat, Praveen Rai, N. A. Vinodini, Anjana Aravind, Thottethodi Subrahmanya Keshava Prasad, K. M. Damodara Gowda

**Affiliations:** 1 Department of Basic Medical Sciences, College of Medicine, University of Sharjah, 27272 Sharjah, United Arab Emirates; 2 Department of Physiology, KS Hegde Medical Academy, Nitte (Deemed to be University), Karnataka Deralakatte, Mangalore, 575018 India; 3 Department of Infectious Diseases & Microbial Genomics, Nitte University Centre for Science Education and Research, Nitte (Deemed to be University), Mangalore, Karnataka 575018 India; 4 Department of Physiology, Kasturba Medical College, Mangalore, Manipal Academy of Higher Education, Manipal, India; 5 Center for Systems Biology and Molecular Medicine, Yenepoya Research Centre, Yenepoya (Deemed to be University), Mangalore, 575018 India

**Keywords:** Health sciences, Medical research

## Abstract

Maternal diet is an essential factor that directly and indirectly regulates fetal growth. Exposure to certain environmental conditions substantially impacts an individual's short- and long-term health. Adipose tissue dysfunction is a worldwide chronic disease caused by improper lipid build-up in adipose tissue leading to obesity. Therefore, it is the need of the hour to invent anti-obesity agents. As a keto–carotenoid, Astaxanthin (AsX) has been shown to have preventive effects against problems associated with obesity. A crucial role in the pathogenesis of obesity has been attributed to dietary polyunsaturated fatty acids. Adipose tissue plays a vital role in maintaining overall body homeostasis. Metabolic dysfunction of white adipocytes forms a critical step in the emergence of insulin resistance and related diseases. Here we aim to investigate the effect of AsX and Docosahexaenoic acid (DHA) supplementation on the proteomic profile of perinatal undernutrition-induced adipose tissue dysfunction in adult life using a rat model. The LC–MS/MS quantitative proteomics enabled us to identify differentially expressed proteins in perinatal undernourished but AsX and DHA-supplemented animal models. Data are available via ProteomeXchange with identifier PXD041772.This study explored biological roles, molecular functions of differentially expressed proteins, and pathways related to adipose tissue dysfunction induced by undernutrition and its effective modulation by AsX and DHA.

## Introduction

Maternal diet is an essential factor that regulates fetal growth, both directly and indirectly, by influencing the development of endocrine systems that govern the foetus’s intake and utilization of nutrients. It also indirectly helps by altering epigenetic profiles and controlling gene expression^1^. In most mammalian species, the perinatal phase is crucial for functional development. As a result, the intrauterine environment has a long-term impact on health following birth^2^. According to Barker (1995), exposure to certain environmental influences may substantially impact an individual's short- and long-term health throughout the crucial stages of development and growth, which is known as the 'Developmental Origins of Health and Disease (DOHaD)' hypothesis^3^. It states, "Alterations in fetal nutrition and endocrine status results in developmental adaptations that permanently change the structure, physiology, and metabolism, thereby predisposing to cardiovascular, metabolic, and endocrine disease in adult life." Obesity is a chronic epidemic metabolic disorder caused by improper lipid build-up in adipose tissue. Obesity is characterized by adipose tissue dysfunction, an abnormal profile of circulatory adipokines, and high levels of other pro-inflammatory substances in the blood^4^. Adult obesity risk is strongly impacted by prenatal and postnatal environmental exposures, notably diet, according to a growing body of evidence. This is the cornerstone of developmental programming^4^. As a result, the programming of the fetus might result from adaptations triggered when the materno-placental nutrition supply falls short of the fetal nutrient requirement, which could be caused by excessive placental inflammation. Many investigations have been conducted in this field to understand the mechanism of the disease condition; also, some interventional studies have given promising results in overcoming obesity-related complications^5^.**T**he current study was designed to evaluate the effect of Astaxanthin (AsX) and Docosahexaenoic acid (DHA) on the proteomic profile of rat adipose tissue that underwent perinatal undernourishment.

AsX is a keto-carotenoid, a secondary metabolite naturally synthesized by several marine crustaceans, fishes, and microalgae, commercially produced from the microalga *Haematococcus pluvialis*. It is a potent scavenger of free radicals, quencher of ROS, and RNS. Also, it exhibits anti-oxidant, anti-inflammatory, anti-diabetic, and anti-lipogenic properties. It has been demonstrated that AsX has some protective effects against obesity-related issues^6^. Dietary polyunsaturated fatty acids (PUFAs) play a significant role in the pathophysiology of obesity. Fish oil-derived n-3 long-chain polyunsaturated fatty acids (n-3PUFAs), including DHA, have been found to improve obesity-associated metabolic disorders ^7–9^.

White adipocytes are responsible for energy storage as triacylglycerols and energy mobilization in mammals as fatty acids. Adipose tissue plays a crucial function in maintaining overall body homeostasis. Lipid metabolism dysfunction, irregular fatty acid biosynthesis, and malfunctioning biological oxidation lead to the onset of obesity^10–12^. Several genomic and proteomic investigations on human and mouse adipose tissue have been conducted previously and showed a significant step in the emergence of insulin resistance and related diseases is metabolic dysfunction in white adipocytes^13^. Though the results of these studies have shed some light on the molecular pathways driving obesity and its accompanying consequences, there is still a paucity regarding the alteration of the proteomic profile of undernutrition-induced obesity in adult life. Therefore, we aim to study the effect of AsX and DHA intervention on the proteomic profile of undernutrition-induced adipose tissue dysfunction in adult life in the rat model.

## Materials and methods

### Chemicals used

Astaxanthin (AsX) was procured from ZENITH NUTRICORP (Edison, USA), and DHA was procured from Carlson Laboratories, Inc (Arlington Heights, USA). Extra virgin olive oil was obtained from ACEITES YBARRA.S.A (SEVILLA, Spain), Lipid profiling kits (TC: cat#11,403,002, TG: cat#11,410,002, HDL: cat#11,414,003) were procured from Agappe Diagnostics, India. Hair cortisol estimation kit (Cat#KLR0828) was obtained from KRISHGEN Biosystems, India. Bovine Serum Albumin (Cat#23,209), Ammonium persulfate (Cat#MKBR5259V), Dithiothreitol (DTT) (Cat# SLBK9476V), Iodoacetamide (IAA) (Cat#SLBP4588V), Sodium Dodecyl Sulphate (Cat#101,639,643), Sodium orthovanadate (Cat#101,645,220), Sodium pyrophosphate (Cat#1,002,102,664), β-glycerophosphate (Cat#101,647,062), Triethylammonium Bicarbonate (Cat# BCBS0954V), Trifluoroacetic Acid (Cat# STBH6629) were procured from Sigma-Aldrich, St. Louis, USA. Pierce™ Bicinchoninic Acid (BCA) Protein Assay Kit (Cat#23,225), Pierce Quantitative Colorimetric Peptide Assay Kit (Cat#23,275), TMT10plex Isobaric Label Reagent Set (Cat#90,406), Acclaim PepMap™ 100 trap column (Cat#10,672,601), PepMap™ RSLC C18 (Cat#10,575,654), 3 M™ Empore™ C18 Extraction Disks (Cat#2215) were purchased from Thermo Fisher Scientific USA. Tris–Hcl (Cat#648,317) was procured from Calbiochem. Bis-Acrylamide (Cat#193,982) was procured from MP Biochemicals. Trypsin (Cat# LS003741) was purchased from Worthington Biochemical Corporation, USA. Formic Acid (Cat#94,318) and Acetonitrile (Cat#1,047,129) were purchased from Merck, Germany. SepPak C18 1 cc cartridges (Cat#0,427,382,554) were obtained from Waters USA, and Acetone (Cat# SJ6SF66920) was procured from EMPARTA.

### Experimental animals and treatments

Eighteen female and six male Albino Wistar rats (200-250 g, 2–3 months old) were procured and kept for breeding. Sample size calculation is based on a mathematical relationship between the following parameters: effect size, variability, significance level, power, and sample size. The current study followed the ARRIVE (Animal Research: Reporting In Vivo Experiments) guidelines. The animals were maintained under controlled conditions of temperature and light in an animal house of the institute and fed standard rat feed and water. All the experiments were performed following the Animal Ethics Committee of our institute (Ref No: KS Hegde Medical Academy/Institutional Animal Ethics Committee, KSHEMA/IAEC/06/2020), and in accordance with ARRIVE guidelines.

### Ethical statement

The below-mentioned treatment protocols and experiments were accomplished based on the guidelines of the Institutional Animals Ethics Committee of our institute.

The experimental animals were divided into three groups of 6 rats each, as Group—I: Control rats (Rats with standard rat feed without any undernourishment & supplementation), Group – II: Peri UN (Perinatally undernourished rats), and Group – III: Peri UN + AsX & DHA (Perinatally undernourished rats supplemented with AsX and DHA).

**Figure 1:**
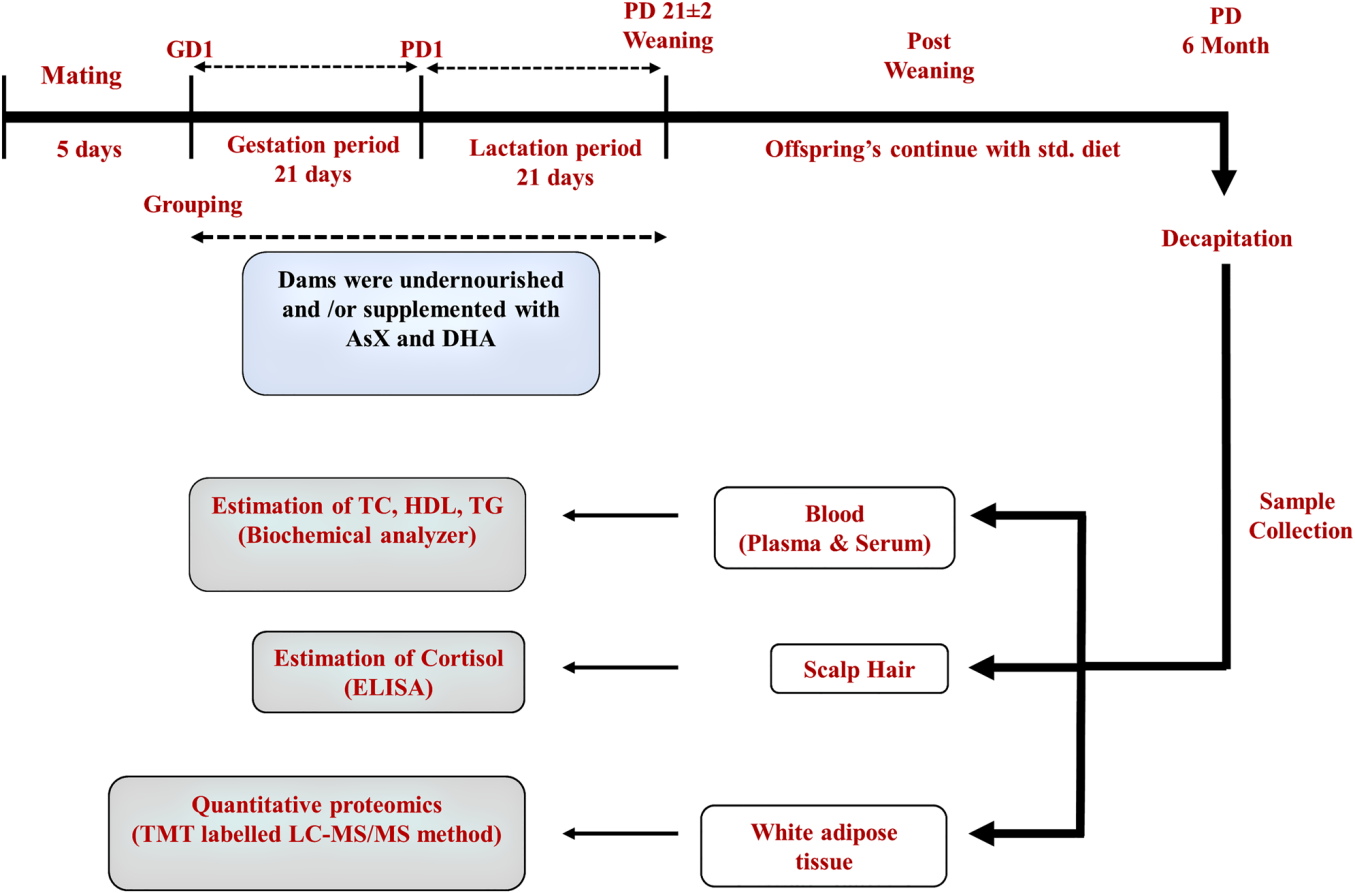
Study design showing the timeline and data collection plan.

Undernourishment of the dams was ensured by reducing 30% of the total food consumption. AsX (24 mg/Kg BW/day) and DHA (500 mg/Kg BW/day) were administered orally during the perinatal period of 42 days. The dosage was selected as per our previous study^14,15^. Olive oil was used to dissolve AsX and DHA. The body weight of the pups was recorded both at the time of birth and six months of postnatal life. The detailed experimental design was represented in Fig. 1.

### Biochemical profiling

After six months, all the animals were sacrificed, and the adipose tissue was collected in a sterile condition. The blood sample was collected in plain test tubes by cardiac puncture. The blood samples were centrifuged at 3000 RPM for 10 min to obtain the serum, and the serum samples were stored at − 20 °C until analysis. The serum lipid (TC, TG, and HDL-C) levels were determined using commercially available kits as per the manufacturer's guidelines (Agappe Diagnostics, India).

### Hair cortisol estimation

Hair samples were collected just before the sacrifice when animals were under anesthesia. Hair was shaved with the help of an electronic razor from the posterior vertex region as close to the scalp as possible. The scissors and other materials were cleaned with 70% alcohol. 100 mg of hair sample was collected from each animal, and samples were wrapped in aluminum foil and stored at room temperature in a dark container. Sample preparation for hair cortisol estimation was performed according to the modified protocol of Bacci et al.^16^. Briefly, the hair sample (100 mg) was washed with water, air dried at RT, placed in a polypropylene tube, and covered with isopropanol (5 mL). The tube was gently mixed (3 min at RT), centrifuged (1500 g for 1 min), and the isopropanol was discarded. The hair sample was again washed with isopropanol, air-dried, and thoroughly pulverized. The pulverized sample (25 mg from each animal was pooled) was placed in a glass vial with 2 ml of methanol and incubated overnight at RT with continuous gentle agitation for steroid extraction. After centrifugation (1500 g for 5 min), the supernatant was collected, transferred into a glass tube, and evaporated to dryness in a vacuum evaporator (37 °C). Hair cortisol was estimated using the commercially available kit (KRISHGEN Biosystems, India) according to the manufacturer's guidelines to confirm undernourishment stress.

### Sample preparation for proteomic analysis—tissue lysis and protein extraction

Ten rat adipocyte tissue samples were retrieved from a -80 °C deep freezer and thawed at room temperature. Tissues were crushed with liquid nitrogen using a mortar and pestle. Once the fine powder of tissue is formed, they were transferred to 1.5 mL microcentrifuge tubes. 4% SDS lysis buffer was added to the samples and subjected to probe–sonication–based protein extraction.

### Protein extraction and estimation

All samples were mixed with 1 mL of lysis buffer (Composition provided in Supplementary Table 1) and incubated at 4 °C for 30 min. Later, the samples were subjected to probe sonication (Q-Sonica, Cole Parmer, India) at 30% amplitude for 2 min on ice. Subsequently, the samples were centrifuged at 10,000 rpm for 20 min at 4 °C. The supernatant was collected and subjected to protein estimation using a Bicinchoninic acid assay (BCA) protein estimation kit following the vendor’s protocol. Once the quantity was estimated, the protein extract was subjected to 10% Sodium Dodecyl Sulphate–polyacrylamide gel electrophoresis (SDS-PAGE) gel to measure the amount of protein and quality check. Followed by the estimation and quality check, equal amounts of protein from each sample were further used for downstream processing. The samples were aliquoted and transferred to chilled acetone for overnight protein precipitation. The next day, the samples were centrifuged at 10,000 rpm for 30 min. The supernatant was removed, and the pellet was dissolved in 50 mM TEABC buffer.

### Reduction, alkylation and protein digestion

To reduce the cysteine bridges, samples were incubated with 10 mM DTT for 30 min at 60 °C. Post reduction, the samples were alkylated with 20 mM of IAA for 20 min under the dark at RT to stop the rearrangement of cysteine bridges. All ten samples, post-reduction, and alkylation were checked for their pH and optimized to pH 8 with 50 mM TEABC and water. Protein digestion was carried out by adding TPCK-Trypsin at a ratio of 1:20 (Enzyme: Protein) and kept for overnight incubation at 37 °C. Digestion efficiency was evaluated by resolving the samples on 10% SDS-PAGE.

### Peptide estimation and normalization: Tandem mass tag (TMT) labelling

Following the trypsin digestion, the peptide samples were subjected to peptide estimation using Peirce Quantitative Colorimetric Peptide Assay (Thermo Fisher Scientific, USA) as per vendors protocol. Based on peptide estimation, the sample volume was normalized to the lowest carrying sample concentration. Peptide samples were labelled using TMT 10plex reagents (Thermo Fisher Scientific, USA) and the reaction was performed as per vendors protocol. Details of the TMT tag used for each sample are provided in Supplementary Table 2. All the samples were pooled together and kept for drying by vacuum evaporation (Savant™ SPD1010 SpeedVac Concentrator, Thermo Fisher Scientific, USA).

### Desalting by waters SepPak C18 1 cc cartridge

The peptides were desalted to remove all the DTT, IAA and SDS present in the samples by Waters SepPak C18 1 cc cartridge. Samples with 200 µg equivalent peptides were diluted ten times using 0.1% formic acid (FA) in water. Desalting was initiated with activation of the C18 bed by passing through 100% acetonitrile and conditioning with 0.1% FA twice. Later, peptide mixture samples were loaded and passed through the C18 cartridge slowly; hence all the peptides will be available for the C18 bind. Washing of C18 cartridges post-sample loading was performed twice using 0.1% FA to remove DTT, IAA, and SDS. Now, peptides bound to the C18 cartridge were eluted slowly by passing 40% ACN in 0.1% FA solution twice. The complete desalting step using C18 StageTips was performed with the help of MicroCL 21R Microcentrifuge at the speed of 500 g under RT.

### Basic reverse-phase liquid chromatography based fractionation

The dried TMT-labeled peptide mixture of rat adipocyte tissues were resuspended with 200 µL of 0.2% tri-fluoro acetic acid (TFA). Meanwhile, StageTip of C18 was prepared, activated, and equilibrated by passing 100 µL of 100% LC–MS grade ACN and 0.2% TFA, respectively. Later, the samples were loaded into the StageTip and passed through twice. The flow-through was collected and stored. The samples were washed with 100 µL of 0.2% TFA twice to remove any salts and surfactants. In contrast, peptides bound to the C18 sorbent were slowly eluted based on their polarity. This was performed by passing 100 µL of 12 elution buffers containing different concentrations of ACN in 10 mM TEABC in water. Finally, the remaining peptides were eluted by passing 100 µL of 80% ACN in 10 mM TEABC in water. All the steps were performed by centrifuging the StageTips at 500 g for 15 min at each stage under RT. The composition of buffers used for C18 fractionation is provided in Supplementary Table 3. After the fractionation, 12 fractions were reduced to 6 by concatenate pooling, and fraction 1, 2, 3, 4, 5, and 6 was pooled with 7, 8, 9, 10, 11, and 12, respectively. All six fractions were allowed to dry in vacuum evaporation (SpeedVac, Thermo Fisher Scientific, USA).

### LC–MS/MS data acquisition

The dried samples were dissolved in 20µL of 0.1% FA. Orbitrap Fusion Tribrid mass spectrometer (Thermo Fischer Scientific, Bremen, Germany) connected to Easy-nLC-1200 nan flow liquid chromatography system (Thermo Fischer Scientific, Germany) for data acquisition. The peptide mixture was loaded onto the Acclaim PepMap™ 100 trap column (75 µm X 2 cm, nanoViper, C18, 3 µm, 100 Å) at a flow rate of 300 nL/min. Peptides were then made to pass through and separate using PepMap™ RSLC C18 (2 µm, 100 Å, 50 µm × 15 cm) analytical column. The peptides bound to the C18 stationary phase of the analytical column were eluted out using two mobile phases, Phase A (0.1% FA in water) and Phase B (0.1% FA in 80% ACN) in gradient mode for 120 min. ESI technique was employed to ionize the peptides under positive ionization mode using EASY-Spray™ Source. The data were acquired in data-dependent acquisition mode. The positively charged ions (peptide precursors) ranging between 400 and 1600 m/z were filtered using quadrupole and trapped in C-trap until AGC reaches 2e5 for 10 ms or either of the first, followed by ion detection in Orbitrap mass analyser under the resolution of 120,000 at 200 m/z. The peptides with charge states 2–7 were considered for analysis, and the dynamic exclusion rate was set to 45 s. Ions were then isolated further using a quadrupole with a 2 Da window based on their intensity and charge state, trapped in C-trap until AGC reaches 1e5 for 200 ms or either of the first, followed by fragmentation using the HCD technique. A 35 ± 3% of NCE was applied, and the fragments ranging between 110 and 2000 m/z were acquired at 60,000.

### Database search

All the MS/MS raw files were searched for peptides and proteins using Mascot and Sequest HT search engines present in Proteome Discoverer 2.2. The proteomic database was searched against the *Rattus norvegicus* proteome database (RefSeq version: 108). Protein sequences were theoretically digested using protease trypsin with maximum two missed cleavages and searched against the LC–MS/MS generated peptide precursor and fragment ions (b and y ions) with 10 ppm and 0.05 Da peptide and fragment mass tolerance, respectively. Modifications such as, TMT, carbamidomethylation at cysteine (C), oxidation at (M), and acetylation (Protein N-termini) were included as fixed and variable modifications. The False Discovery Rate (FDR) was calculated using the Percolator node. To calculate the FDR, the decoy database was generated by reversing the amino acid sequences at the peptide and protein levels. The PSMs identified with FDR less than 1% (q-value < 0.01) were considered accurate matches. Details of database search parameters used in Proteome Discoverer 2.2 are provided in Supplementary Table 4.

### Statistical analysis

The results acquired from Proteome discoverer software were used for further data analysis. The normalization of the abundance values at the protein level were carried out using median normalization. The fold change was calculated using the normalized abundance values for the samples corresponding to their control. The proteins with a fold change > 1.3 were considered overexpressed and < 0.67 as down-regulated. The significant proteins were identified with the *p*-value < 0.05 calculated using the Student’s t-test.

### Functional enrichment analysis

Proteins were classified and categorized based on Gene Ontology analysis for cellular components and molecular functions using DAVID 8.6 functional annotation tool (https://david.ncifcrf.gov/summary.jsp) and pathway analysis using Reactome 83 (https://reactome.org/) tool. The interaction network analysis of proteins based on their biological process was carried out using the Cytoscape tool version 3.9.1. The heat map for the differentially expressed proteins and the bubble plot for the representation of cellular component analysis of the proteins was generated using R studio version 3.6.0. (https://www.R-project.org/) The sankey diagram was generated using an online Sankey generator (http://sankey-diagram-generator.acquireprocure. com/).

## Results

### Perinatal undernutrition stress induces adipose tissue dysfunction

**Figure 2:**
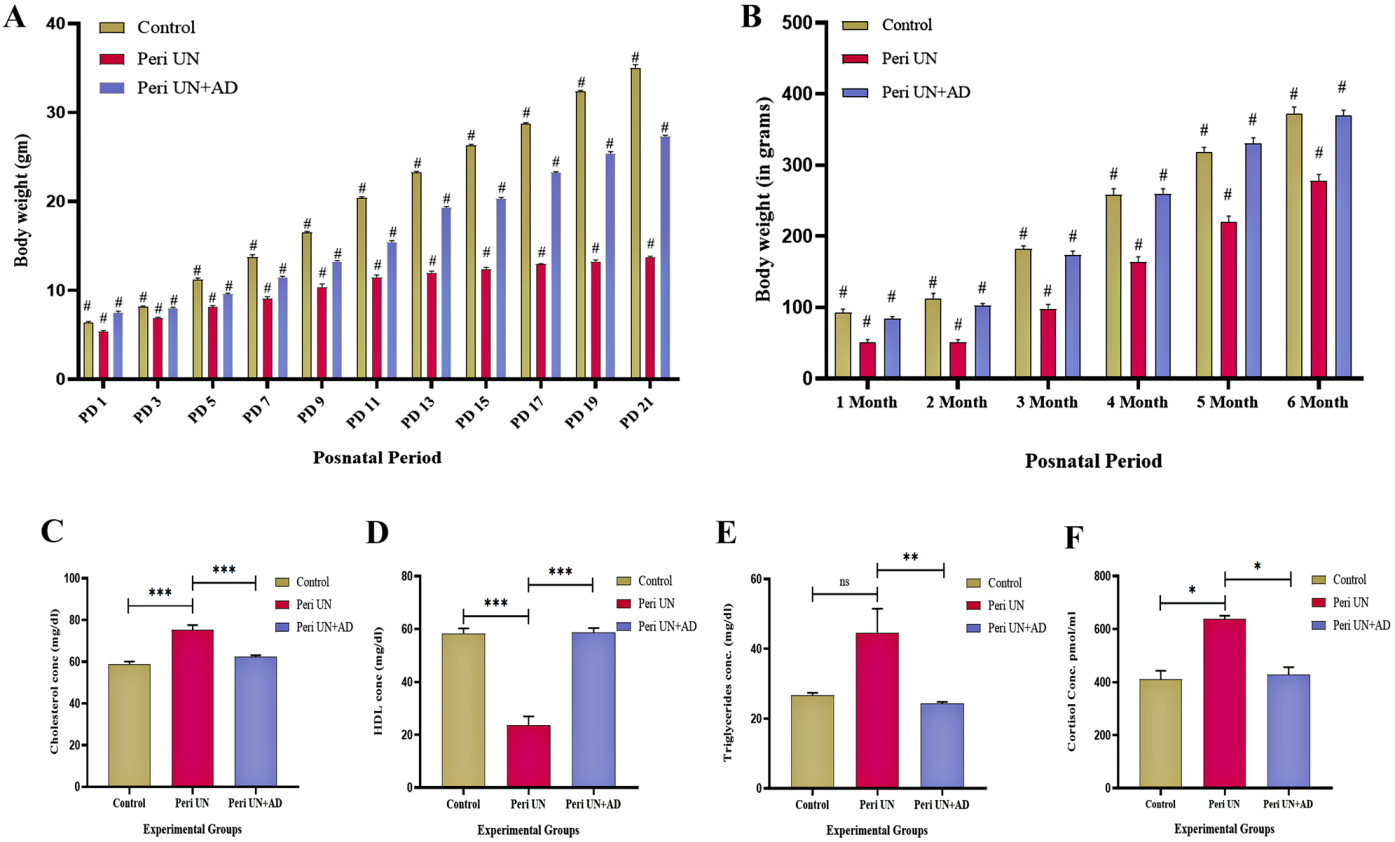
(**A**) Pups body weight: postnatal day 1-21. (**B**) Pups body weight: 1-6 month (n=4). (**C**) Serum cholesterol level (n=4). (**D**) Serum triglycerides level (n=4). (**E**) Serum HDL level (n=4). (**F**) Hair cortisol level (n=4).

We aimed to establish perinatal undernutrition stress-induced obesity and to evaluate the effect of AsX and DHA supplementation in the rat model. To achieve this, nine healthy female albino Wistar rats were randomly divided into three groups (3 in each group, housed in 3 different polypropylene cages). A male rat was introduced into every cage and allowed for breeding. All the animals were maintained under a 12 h light and dark cycle and controlled temperature conditions. Once the pregnancy gets confirmed by observing vaginal plug formation, male rats were removed from breeding cage. Then, each group of rats underwent respective treatments for a perinatal period of 42 ± 4 days (Prenatal 21 ± 2 days, postnatal 21 ± 2 days). Treatment groups were divided, as explained in the methods section. Perinatal undernutrition was ensured by giving only 70% of the actual food consumption to the dams^15^. Olive oil was used for dissolving AsX and DHA, and this formulation was administered orally. Body weights of the pups were noted every alternate day till 21 days of their postnatal life (Fig. 2A). Later, the body weight was recorded monthly until the pups reached six months of adult age (Fig. 2B). Two-way ANOVA followed by Tukey's multiple comparisons test was used to find the significant statistical difference between the groups. A significant difference was considered if the p-value was < 0.05. The birth weight of the perinatally undernourished group was significantly reduced (*p* < 0.0001) compared to perinatally undernourished with AsX & DHA -supplemented group pups (Fig. 2A). Pups belong to perinatally undernourished group showed significantly lowered body weight (*p* = 0.0001) compared to control and Peri UN + AsX & DHA-supplemented group consistently from postnatal day 3 to 21. This confirms that perinatal undernourishment caused decrease in body weight, which was ameliorated by AsX and DHA supplementation.

### Biochemical parameters

Serum total cholesterol, triglycerides, and HDL-C levels were determined using commercially available kits per the manufacturer's guidelines (Agappe Diagnostics, India). One way-ANOVA or non-parametric test followed by Tukey's multiple comparisons test was used to find the significant statistical difference between the groups. Serum cholesterol level was significantly higher in the perinatally undernourished group compared to the control (*p* = 0.0001) and Peri UN + AsX & DHA supplemented (*p* = 0.0007) group, as shown in Fig. 2C. Serum HDL level was significantly lower in the perinatally undernourished group when compared to the control (*p* < 0.0001) and Peri UN + AsX & DHA supplemented group (*p* < 0.0001) as represented in Fig. 2D. Serum triglyceride level was found to be significantly elevated in the perinatally undernourished group compared to Peri UN + AsX & DHA supplemented group (*p* = 0.001, Fig. 2E). Therefore, it was evident that perinatal undernourishment caused an abnormal lipid profile, showing the indications of future obesity, whereas AsX and DHA supplementation ameliorated the altered lipid profile.

To ensure perinatal undernourishment, we have estimated long-time hair cortisol levels among experimental groups. The perinatally undernourished group showed a significantly increased amount of cortisol compared to the control (*p* = 0.016) and Peri UN + AsX & DHA supplemented group (*p* = 0.021, Fig. 2F).

### Quantitative proteomics highlights differentially regulated proteins

**Figure 3:**
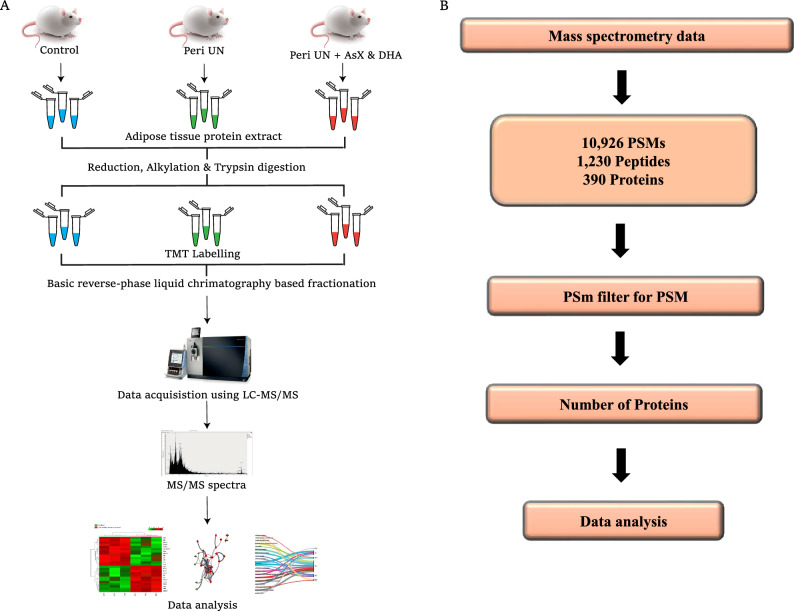
Summary of proteomics analysis (**A**) Schematic of workflow employed for proteomics data acquisition. Labels: VC- vehicle control; Peri UN – Perinatally undernourished; Peri UN + AsX & DHA- Perinatally undernourished + Astaxanthin and DHA supplemented. (**B**) Schematic of the workflow used for proteomics data analysis.

Quantitative proteomics was carried out to identify the altered proteins with perinatal undernutrition and the restoration of those by AsX and DHA treatment. The data was acquired for the biological triplicates, as technical triplicates each (Fig. 3A). The workflow used for filtering proteins based on their identification from the independent replicates is outlined in Fig. 3B. When MS/MS data compared to the database, we found 10,926 PSMs from the 4, 82,393 tandem mass spectrometry (MS/MS) spectra of *Rattus norvegicus*. Proteins often seen in biological replicates were taken into account. While identifying these PSMs' 1,230 non-redundant peptides, leading to the identification of 390 proteins, which were then employed for further study (Supplementary Information Table).

**Figure 4:**
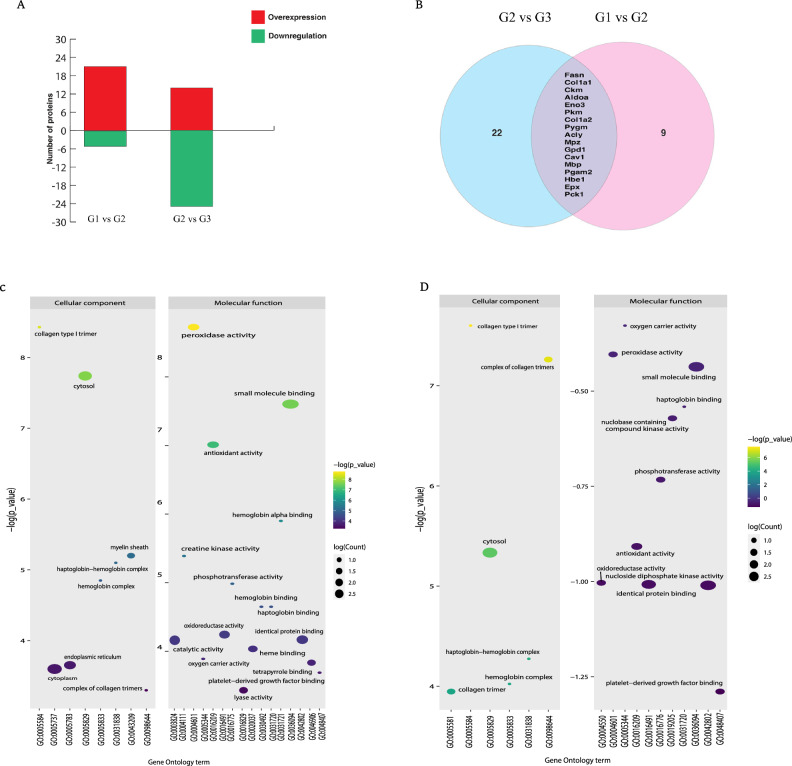
(**A**) Bar graph showing differentially regulated proteins among all the groups. (**B**) Venn diagram showing the restoration of proteins with AsX and DHA treatment. (**C**) Bubble plot showing and molecular functions of differentially expressed proteins in Control vs Peri UN group and (**D**) Peri UN + AsX & DHA supplemented group.

The FC of protein expression was calculated with respect to the control group and the perinatally undernourished group. A cut-off Fold Change of > 1.3 for overexpression and < 0.67 for the down regulation of proteins was considered biologically significant, with an adjusted *p*-value of q ≤ 0.05 selected for statistical significance. Figure 4A explains the number of differentially expressed proteins among all three experimental groups. Undernutrition stress highlighted 26 differentially expressed proteins (FC > 1.3 or < 0.67), which includes 21 significantly overexpressed and five down regulated proteins (q ≤ 0.05) when compared to the control (Table 1). AsX and DHA treatment substantially regulated 39 proteins (q ≤ 0.05) in the undernourished group, which included the restoration of 17 dysregulated proteins due to undernutrition stress by AsX and DHA supplementation (Fig. 4B).

**Table 1:** Differentially expressed proteins Peri UN v/s Control.

Protein	Accession no	FC value	*P* value
Overexpressed proteins			
Fasn	NP_059028.2	2.276	0.010
Hbb	NP_150237.1	1.414	0.046
Col1a1	NP_445756.1	2.284	0.020
Actc1	NP_062056.1	4.388	0.049
Ckm	NP_036662.1	5.435	0.005
Aldoa	XP_038950008.1	2.294	0.023
Eno3	XP_006246661.1	2.931	0.020
Car3	NP_062165.2	1.492	0.015
Pkm	XP_006243252.1	1.781	0.008
Col1a2	NP_445808.2	1.520	0.014
Mdh1	NP_150238.1	1.594	0.004
Ces1d	NP_579829.3	1.649	0.002
Pygm	NP_036770.1	2.586	0.000
Acly	NP_058683.2	2.174	0.035
Prdx1	NP_476455.1	1.383	0.044
Mpz	NP_001300997.1	3.607	0.035
Gpd1	NP_071551.2	1.645	0.004
Cav1	NP_113744.2	1.740	0.026
Mbp	XP_006255059.1	3.210	0.040
Pgam2	NP_059024.1	1.974	0.021
Ckb	NP_036661.3	4.195	0.049
Downregulated Proteins			
Gsn	NP_001004080.1	0.681	0.001
Hbe1	NP_001008890.1	0.642	0.009
Epx	NP_001100507.1	0.521	0.000
Pck1	NP_942075.1	0.638	0.026
Atl3	XP_006230874.1	0.557	0.002

### Classification of identified proteins

Gene ontology annotations were used to classify the proteins based on their intracellular compartmentalization, molecular function, and biological processes to understand the relevance of the differentially regulated proteins in undernutrition stress. Differentially regulated proteins identified in the perinatally undernourished group (Fig. 4C). These proteins were mainly localized in the cytoplasm, cytosol, and endoplasmic reticulum, followed by a myelin sheath, complex of collagen trimmers and haptoglobin − hemoglobin complex. Also these proteins are involved in molecular functions such as lyase activity, catalytic activity, heme binding, tetrapyrrole binding, oxido-reductase activity, antioxidant activity, small molecule binding, peroxidase activity, etc. The proteins regulating these functions were further shortlisted to understand the effect of undernutrition stress and their rescue by AsX and DHA supplementation (Fig. 4D).

### Perinatal undernutrition stress dysregulates proteins involved in adipose tissue functions

**Figure 5:**
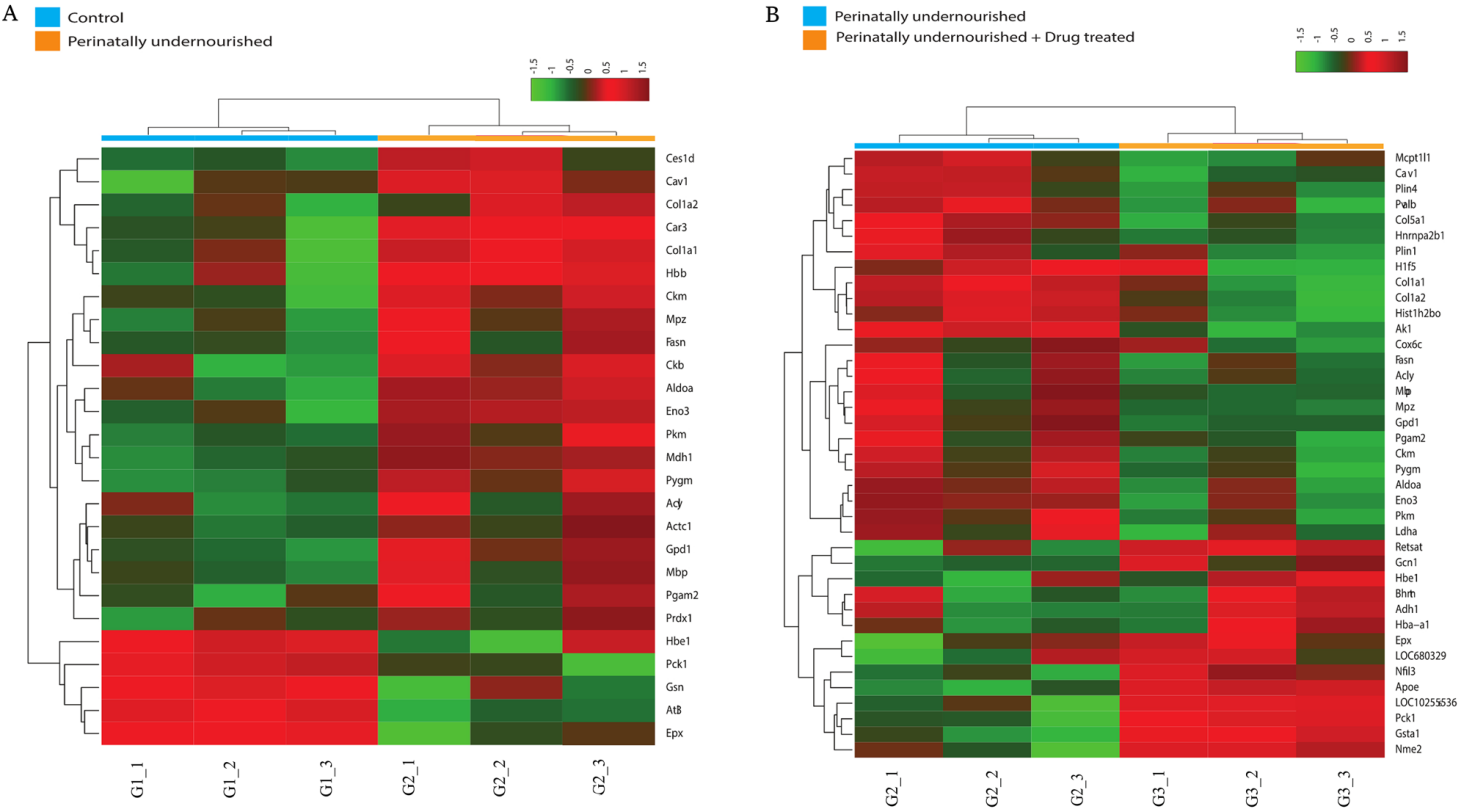
(**A**) Heat map showing differentially expressed proteins with perinatal undernutrition stress compared to control and (**B**) Differentially expressed proteins with Peri UN compared to AsX & DHA supplemented group.

Proteomics data was utilized to acquire deeper knowledge into undernutrition stress-induced adipose tissue dysfunction. Undernutrition stress in perinatally undernourished group resulted in 21 overexpressed (FC ≥ 1.3, q ≤ 0.05) and five down-regulated proteins (FC ≤ 0.67, q ≤ 0.05) when compared to control (Table 1, Fig. 5A).

The perinatally undernourished group showed overexpression of 2 major enzymes related to the onset of obesity. They are, (1) Fatty acid synthase (FASN), which plays an important role in fatty acid metabolism and biosynthesis. (2) Fructose-bisphosphate aldolase A (Aldoa), which is a key glycolytic pathway enzyme, responsible for developing insulin sensitivity. In addition, the perinatally undernourished group showed under expression of one major enzyme, Phospho-enolpyruvate Carboxykinase (Pck1 cytosolic form). Pck1 is involved in regulating the triglyceride/fatty acid cycle.

### Astaxanthin and DHA restores proteins involved in undernutrition stress induced adipose tissue dysfunction

We have compared the AsX and DHA supplemented group with the perinatally undernourished group for differentially regulated proteins, which have.been highlighted in a heat map (Fig. 5B). Those over expressed proteins (FASN & Aldoa) involved in vital functions of adipose tissue gets restored in AsX and DHA supplementation group (Table 2). Moreover, we found down regulation of L-lactate dehydrogenase A chain isoform X1(LDHA), which is involved in aerobic/anaerobic glycolysis. Also, glycerol-3-phosphate dehydrogenase (GPD-1) got down regulated, an essential enzyme for synthesizing adipose tissue triacylglycerols (TAG).

**Table 2:** Differentially expressed proteins Peri UN + AD v/s Peri UN.

Protein	Accession No	FC value	*P* value
Overexpressed proteins			
Hba-a1	NP_037228.1	1.531	0.031
Apoe	NP_620183.2	1.378	0.020
Bhmt	NP_110477.1	1.554	0.046
Hbe1	NP_001008890.1	1.868	0.008
Gsta1	NP_113697.1	1.712	0.042
Epx	NP_001100507.1	1.641	0.003
LOC680329	XP_038944887.1	1.377	0.026
Retsat	NP_659552.1	1.472	0.042
Adh1	NP_062159.3	1.826	0.033
Nme2	NP_114021.2	1.476	0.000
LOC102555366	XP_038943779.1	2.714	0.003
Nfil3	XP_006253732.1	1.862	0.016
Pck1	NP_942075.1	1.935	0.000
Gcn1	NP_001162135.1	1.485	0.012
Downregulated proteins			
Fasn	NP_059028.2	0.445	0.010
Col1a1	NP_445756.1	0.476	0.013
Ckm	NP_036662.1	0.259	0.009
Aldoa	XP_038950008.1	0.511	0.040
Eno3	XP_006246661.1	0.450	0.042
Pkm	XP_006243252.1	0.550	0.007
Ldha	XP_006229294.1	0.666	0.043
Col1a2	NP_445808.2	0.402	0.000
Plin1	NP_001295074.1	0.671	0.036
Hist1h2bo	NP_001099584.1	0.678	0.015
Pygm	NP_036770.1	0.430	0.001
Acly	NP_058683.2	0.474	0.033
Mpz	NP_001300997.1	0.315	0.043
Gpd1	NP_071551.2	0.670	0.010
Pvalb	XP_006241991.1	0.327	0.025
Cav1	NP_113744.2	0.614	0.001
Plin4	XP_006244434.1	0.552	0.006
Mcpt1l1	NP_001264597.1	0.568	0.004
Ak1	XP_038960159.1	0.527	0.002
Mbp	XP_006255059.1	0.343	0.048
H1f5	NP_001102887.1	0.494	0.027
Pgam2	NP_059024.1	0.453	0.010
Col5a1	NP_604447.2	0.580	0.005
Hnrnpa2b1	NP_001098083.2	0.663	0.011
Cox6c	NP_062233.2	0.733	0.048

### Astaxanthin and DHA modulate pathways involved in dysregulated fatty acid metabolism

**Figure 6:**
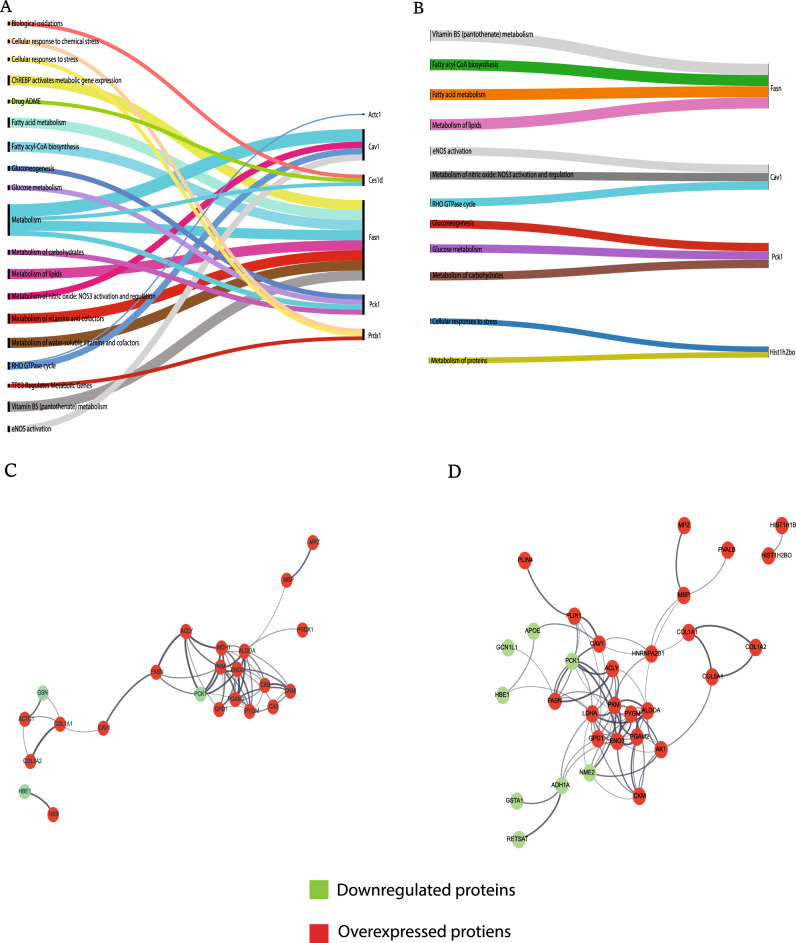
Sankey diagram showing the pathways associated with the differentially regulated proteins in (**A**) Control vs Peri UN group and (**B**) Peri UN vs Peri UN + AsX & DHA supplemented group (**C**) The interaction network analysis of proteins based on its biological process in Control vs Peri UN group and (**D**) Peri UN vs Peri UN + AsX & DHA supplemented group.

Protein dynamics greatly influence the regulation of various cellular processes. Significant pathways were identified from the pathway enrichment analysis (Fig. 6A). They were then narrowed down to essential pathways, such as biological oxidation of very long chain fatty acids, fatty acid metabolism, fatty acyl CoA biosynthesis, cellular response to stress, lipid metabolism, etc.

In the current study, we investigated and confirmed the dysregulated proteins caused by perinatal undernutrition stress are involved in the functions of adipose tissue. These differentially expressed proteins involved in lipid / fatty acid metabolism leading to adipose tissue dysfunction. Further, it leads to the onset of obesity. In this study, we also demonstrated that AsX and DHA supplementation repaired the pathways involved in dysregulated fatty acid metabolism by suppressing the over-expressed proteins involved in the lipogenic pathway in adipose tissue (Fig. 6B).

### Biological processes regulated by Astaxanthin and DHA:

Gene Ontology Biological Process (GO_BP) annotation for the whole co-expression network was performed. The size of the nodes reflects the number of proteins involved in the corresponding process and the colour gradient of the node represents the statistical significance of each term. Different node colours represent other functional groups. The name of each group is given by the essential term of the group. The nodes are grouped by the similarity of their associated genes. In this category, the proteins were mainly associated with the lipid metabolic process, lipid biosynthetic process, and response to lipid and fatty acid biosynthetic process (Fig. 6C, D).

## Discussion

The current study proved the beneficial effect of AsX and DHA to minimize the adverse effects of perinatal undernutrition stress in the rat model. Humans are exposed to various environmental insults, including nutrition as a significant component throughout their lives, which causes alterations in the whole body's composition and functions. The main aim of the current study was to establish the mechanism of perinatal undernutrition stress-induced adipose tissue dysfunction. To achieve this, we prepared and standardized a rat model by feeding the dams with 70% of their food consumption. The pups born to perinatally undernourished dams were used as experimental animals.

The hair cortisol concentration (HCC) of the animals in the various experimental groups was measured to confirm and validate the undernutrition stress, as measuring HCCs for stress evaluation has many advantages. The measurement of cortisol in hair serves as a retrospective biomarker of increased cortisol production reflecting exposure to various life stressors^17^. The current study revealed a significant increase in HCC in the perinatally undernourished group compared to the control and Peri UN + AsX & DHA-supplemented group. This suggests that animals have experienced undernutrition stress, and AsX and DHA may have the capacity to ameliorate the fatal effect of perinatal undernourishment stress.

Several studies have investigated the relationship between cortisol levels and obesity. In the present study, we observed an increase in the cortisol level in perinatally undernourished animals and exhibited an increase in the obesity markers during their adult life. Obesity and related metabolic problems are brought about by prolonged exposure to high-circulation amounts of glucocorticoids. Glucocorticoid receptors are anti-inflammatory in action due to their non-genomic and genomic property. Genomic activity is mediated by nuclear receptor translocation and activation of vital anti-inflammatory genes (MKP-1, IL-10, annexin-1) and repression of proinflammatory genes (TNF-α, IL-6, IL-1β). Therefore, the impaired function of the glucocorticoid receptor is likely a mechanism for this chronic inflammation in obesity^18^. Due to the physiological mechanism whereby glucocorticoids stimulate the maturation of preadipocytes into mature adipocytes, increasing cortisol concentrations have been causally linked to fat storage and weight gain^19^. Stress-related cortisol levels affect adipocyte biology and cause significant weight gain, potentially implicating a crucial factor in the development of obesity.

The body weight was decreased during initial postnatal days of pups from day-1 to 21 in 6-month-aged experimental animals; surprisingly obese phenotype was not observed in this study. Nevertheless, we observed an obese phenotype caused by perinatal undernourishment stress in 1-year and 2-year-old rats in another study conducted by our group (unpublished data). Though we did not observe obese phenotype among the experimental animals, the proteomics results showed adipose tissue dysfunction in perinatally undernourished animals. It was evidenced by the differential regulation of key proteins involved in adipogenesis and lipid metabolism. This indicated that AsX and DHA ameliorate dysregulated fat metabolism by regulating the proteins involved.

The current study employed proteomics approach to demonstrate that perinatal malnutrition induced adipose tissue dysfunction. Adipose tissues can quickly and dynamically change through variations in the quantity and size of adipocytes in response to changes in metabolism. De novo lipogenesis, adipogenesis, apoptosis, and thermogenesis modify adipose tissue function^10^. The primary characteristics of obesity include growth and de novo lipogenesis. Lipogenesis promotes fatty acid production and increases adipocyte size and hypertrophy. Adipogenesis generates new cells and increases adipocyte number, and hyperplasia leads to an aberrant deposition of adipose tissues^20^. Adipocyte development from precursor cells is known as adipogenesis. It was well acknowledged that adipogenesis was governed by a transcriptional cascade of precursor cells and involves the accumulation of fat in lipid droplets, which induces differentiation and maturation^11^.

The pathway and Gene Ontology analysis identified differentially expressed proteins and elucidated the underlying mechanisms of perinatal undernourishment-induced adipose tissue dysfunction. Overexpression of fatty acid synthase (FASN), fructose-bisphosphate aldolase A (AldoA), pyruvate kinase isoform M2 (PKM2), and carboxylesterase 1D (Ces1D) was observed in the undernourished animals compared to the control group. They play a crucial role in fatty acid metabolism, lipid metabolism, fatty acyl-CoA biosynthesis, metabolism of glucose, RHO-GTPase cycle, etc. It was well reported that obesity develops due to the up-regulation of the mediators of the lipogenic pathway and its activity in adipose tissue^21^. The FASN gene was discovered to be a potential gene for determining body fat since it is a key enzyme in lipogenesis. Increased FASN gene expression in adipose tissue is associated with the accumulation of visceral fat, decreased insulin sensitivity, and a rise in fasting insulin levels indicating a critical role for lipogenic pathways in the causal relationship between the harmful effects of excessive energy intake and the onset of obesity^12,21,22^.

It was reported that the etiology of obesity includes the up-regulation of AldoA. In the glycolytic route, AldoA breaks down F-1,6-BP to create GA3P and DHAP, the primary building blocks of methylglyoxal (MG), highly reactive metabolites produced by mammalian cells^23,24^. They asserted that insulin increases the synthesis of MG in insulin-sensitive adipocytes by up-regulating AldoA. This process may contribute to the emergence of insulin resistance, which eventually results in obesity^25^.

In addition, we noticed an increase in the activity of pyruvate kinase PKM isoform X2 (PKM2). PKM2 is an essential enzyme that catalyses the conversion of phosphoenolpyruvate to pyruvate at the end of glycolysis. PKM2 controls gene expression in the nucleus and phosphorylates several crucial proteins that control vital cell signalling pathways and their metabolic function in glycolysis^26^.

It was reported that HIF-1 (a gene involved in the pathogenesis of metabolic disorders) expression is increased due to increased PKM2 expression via the PI3K/mTOR pathway^27^. It has been shown that HIF-1 increases obesity-related inflammation, inhibits insulin signaling, and promotes angiogenesis. To reduce the adipose tissue hypoxia brought on by adipocyte expansion, PKM2 influences HIF-1 expression, implying that PKM2 controls the insulin pathway and inflammation linked to obesity^28^. In contrast, Iqbal et al.^29^ reported that PKM2 activity reduction promotes lipid synthesis.

In the current study, we found the up-regulation of Ces1D, which is consistent with the study conducted by Jernas et al.^30^. According to them, adipose tissue from obese people expressed Ces1D much more than lean subjects. Ces1D expression in adipose tissue was linked to metabolic factors such as total cholesterol, LDL cholesterol, and HDL cholesterol. They also noticed that obese participants' Ces1D expression was reduced due to diet-induced weight loss. However, the mechanisms are unclear.

Moreover, we found the up-regulation of the Caveolin-1 isoform (Cav-1) in the perinatally undernourished group. The plasma membrane contains caveolae, a form of lipid raft, which are flask-shaped invaginations extending 50–100 nm into the cytoplasm. Most cell types have caveolae, but adipocytes have most, making up 30% of the plasma membrane surface area^31^. Cav-1 isoform is responsible for caveolae production in adipocytes^32^. The 21–24 kDa integral membrane proteins known as caveolins act as scaffolding to draw in various signaling molecules. It has been suggested that caveolae have a role in endocytosis, substrate transport, signal transduction, and membrane traffic. In 2010, Fernandez-Real et al. investigated the involvement of the Cav-1 protein in the development of obesity^33^. They discovered that mature adipocytes had much greater CaV-1 expression than stromal vascular cells. CaV-1 gene expression was significantly correlated with fasting triglycerides in adipocytes from subcutaneous adipose tissue. Despite a considerable rise in FAS gene expression, CaV-1 gene expression remained unchanged primarily throughout human pre-adipocyte differentiation from lean or obese people. Hence, they concluded that in obese individuals, lower CaV-1 gene expression is concurrently associated with higher triglycerides and decreased lipogenic gene expression. We demonstrated the down-regulation of CaV-1 gene expression in the group treated with AsX and DHA, aligned with Fernandez-Real et al. study^33^.

In the current study, we observed the down-regulation of phosphor-enol-pyruvate carboxykinase (Pck1) compared to other proteins. The role of the Pck1 gene in high-fat diet (HFD)-induced obesity in transgenic mice was investigated by Franckhauser et al. in 2006^34^. They reported that the transgenic mice fed with HFD acquired twice as much body weight as a control because of overexpression of the Pck1 gene in adipose tissue. This confirmed the development of obesity in the adult life of rats that underwent perinatal undernutrition.

In AsX and DHA-treated group, all previously mentioned proteins contributing to adipose tissue dysfunction were down-regulated. Feng et al.^35^ reported that, an increased lactate generation, a L-lactate dehydrogenase A (LDH-A) product, in adipocytes in different metabolic disorders including obesity. This alarming signal drives adipose tissue macrophage (ATM) polarization to an inflammatory state. But, to our surprise, we observed the down-regulation of L-lactate dehydrogenase A isoform X1 (LDH-A). Rats were protected from obesity-related glucose intolerance and insulin resistance by adipocyte-selective deleting of the enzyme lactate dehydrogenase A (LDH-A), which converts pyruvate to lactate. This protection was accompanied by a lower percentage of inflammatory ATM and decreased production of pro-inflammatory cytokines like interleukin 1 (IL-1). It suggests that AsX and DHA are involved in the restoration of the expression of these genes. This proved the anti-obesity effect of AsX and DHA in a rat model of perinatal undernutrition stress.

In conclusion, to the best of our knowledge, this is a pioneer proteomics report of perinatally undernourished rat adipose tissue dysfunction by identifying 390 different proteins with a few differentially expressed proteins. Furthermore, we demonstrated that FASN, Aldoa, PkM2, and Ces1D are potentially overexpressed proteins. AsX and DHA treatment significantly ameliorated the perinatal undernutrition stress-induced differential expression of proteins in adipose tissue. The relevance of other differentially expressed proteins deserves further research.

Limitations of the current study was being identified a limited number of proteins. The probable reason for the less identification of proteins might be the use of probe–sonication–based protein extraction. Heat generated during this process might have caused degradation of proteins in the sample. Another reason might be,  instead of acetone precipitation, methanol-chloroform precipitation during sample processing might have helped us remove complete fat content from the sample, leaving only the proteins behind. Due to financial and laboratory limitations, we could not compare and correlate with clinical examples. Hence the differential outcomes in animal data and clinical samples led to another limitation of our investigation. Therefore, additional clinical sample proteomics analysis is warranted to dig deeper into knowledge in this regard.

## Supplementary Information

Supplementary Figures.

Supplementary Tables.

## Data Availability

The mass spectrometry proteomics data have been deposited to the ProteomeXchange Consortium via the PRIDE [1] partner repository with the dataset identifier PXD041772.

## Abbreviations

ACN: Acetonitrile
AsX: Astaxanthin
BCA: Bicinchonic acid
BSA: Bovine serum albumin
DDA: Data dependent acquisition
DHA: Docosahexaenoic acid
DOHaD: Developmental origins of health and disease
DTT: Dirhiothreitol
FASN: Fatty acid synthase
FC: Fold change
GAPDH: Glyceraldehyde-3-phosphate dehydrogenase
HDL: High density lipoprotein
HCC: Hair cortisol concentration
IAA: Iodoacetamide
LC MS/MS: Liquid chromatography tandem mass spectrometry
L-FABP: Liver- fatty acid binding protein
n-3PUFAs: N-3 long-chain polyunsaturated fatty acids
PeriUN + AsX & DHA: Perinatally undernourished rats supplemented with Astaxanthin and Docosahexaenoic acid
Peri UN: Perinatally undernourished rats
PSMs: Peptide-spectrum matches
PUFAs: Polyunsaturated fatty acids
RNS: Reactive nitrogen species
ROS: Reactive oxygen species
RT: Room temperature
SDS-PAGE: Sodium dodecyl sulphate poly acrylamide gel electrophoresis
TC: Total cholesterol
TEABC: Triethyl ammonium bicarbonate buffer
TG: Triglycerides
TMT: Tandem mass tag
