# Supplementary material for: Astaxanthin and DHA supplementation ameliorates the proteomic profile of perinatal undernutrition-induced adipose tissue dysfunction in adult life
Source: Sci Rep. 2023 Jul 29;13:12312. doi: 10.1038/s41598-023-38506-x (PMC10387058; doi:10.1038/s41598-023-38506-x)
Supplement: Supplementary file 1 — Supplementary Figures. [file 41598_2023_38506_MOESM1_ESM.pdf]

Protein estimation:

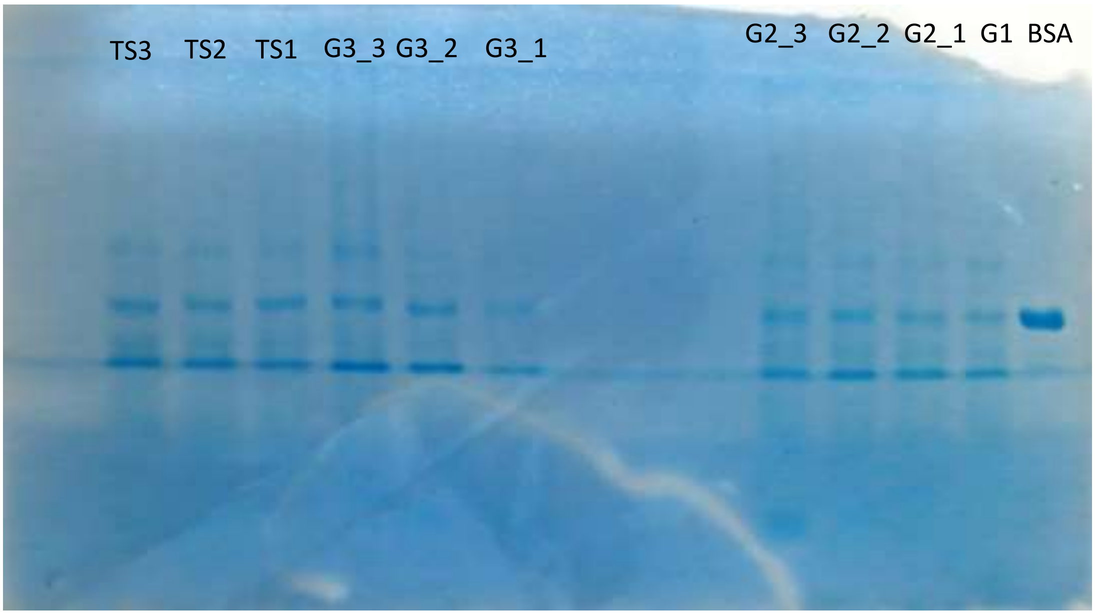

Image 1: Protein estimation by SDS-PAGE

* 2µg BSA was loaded as control

Proteome search result:

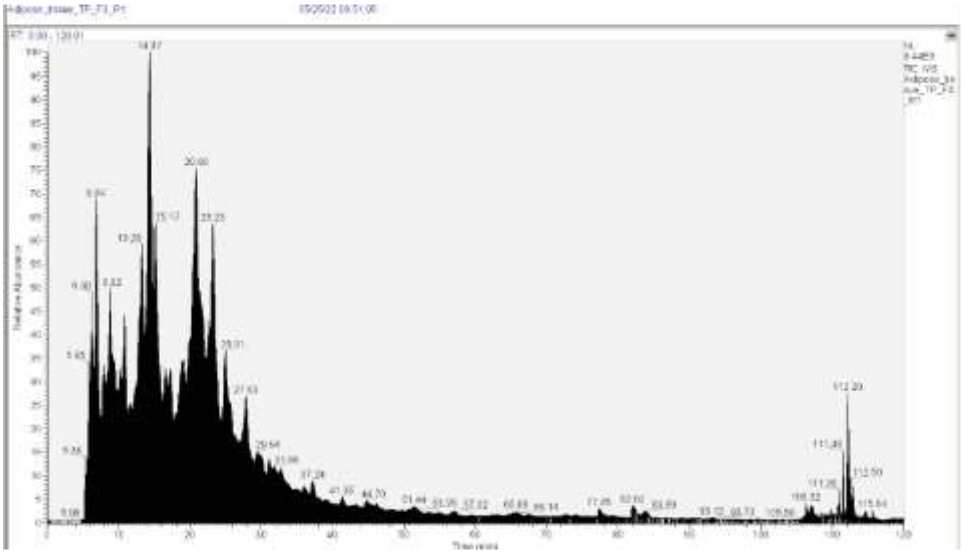

Image 2: 120min chromatogram of fraction 3 of rat adipose tissue sample
