# Supplementary material for: Astaxanthin and DHA supplementation ameliorates the proteomic profile of perinatal undernutrition-induced adipose tissue dysfunction in adult life
Source: Sci Rep. 2023 Jul 29;13:12312. doi: 10.1038/s41598-023-38506-x (PMC10387058; doi:10.1038/s41598-023-38506-x)
Supplement: Supplementary file 2 — Supplementary Tables. [file 41598_2023_38506_MOESM2_ESM.pdf]

Astaxanthin and DHA supplementation ameliorates the proteomic profile of perinatal undernutrition-induced adipose tissue dysfunction in adult life

[1]Anu V Ranade¶, [2]Pramukh Subrahmanya Hegde¶, [2]Megha Agni Bhat, [3]Praveen Rai, [4]Vinodini N A, [5]Anjana Aravind, [5]Thottethodi Subrahmanya Keshava Prasad, [2]Damodara Gowda K M*

[1]Department of Basic Medical Sciences, College of Medicine, University of Sharjah-27272, Sharjah, United Arab Emirates. [2]Department of Physiology, K.S.Hegde Medical Academy, Nitte (Deemed to be University), Deralakatte, Mangalore-575018, Karnataka, India. [3]Nitte University Centre for Science Education and Research, Nitte (Deemed to be University), Mangalore, 575018, Karnataka, India. [4]Department of Physiology, Kasturba Medical College, Mangalore, Manipal Academy of Higher Education, Manipal, India. [5]Center for Systems Biology and Molecular Medicine, Yenepoya Research Centre, Yenepoya (Deemed to be University), Mangalore, 575018, India.

***Correspondig author:** Dr, Damodara Gowda K M, E-mail: dr_damodar@nitte.edu.in

¶ Equal contribution

Suppl. Table 1: Composition of lysis buffer

Materials	Stock	Amount (1mL)	Amount (30mL)
SDS (4% v/v)	10% Stock	100 µL	3000 µL
Sodium orthovanadate	100 mM	10 µL	300 µL
Sodium pyrophosphate	125mM	20 µL	600 µL
β-glycerophosphate	1 M	1 µL	30 µL
TEABC	1M	5 µL	60 µL

Suppl. Table 2: Details of TMT tag

Based on peptide estimation, the sample volume was normalized to lowest concentration carrying sample. The TMT reaction was performed as per vendor protocol. Breifly, equal volumes of TMT labels dissolved in 100% anhydrous ACN were added, vortexed for 30 secs, centrifuged at 8,000 rpm for 2 min and kept for incubation at room temperature for 1 hour. After 1 hour of incubation, 2 µL of sample from each were aspirated, quenched by adding 1 µL of 5% hydroxylamine and pooled. The quenching reaction was performed at RT for 15

min. Please find the table 2 for details of the TMT and samples. All the samples were pooled together and kept for drying by vacuum evaporation (Savant™ SPD1010 SpeedVac Concentrator, Thermo Scientific) at RT.

Details of TMT tag used for each sample

TMT tag	Sample ID
126	G1
128C	G2_1
129N	G2_2
129C	G2_3
130N	G3_1
130C	G3_2
131	G3_3

Suppl. Table 3: Composition of buffer used for SCX fractionation

Fraction number	Acetonitrile (%)	Volume mobile phase A (µl)	Volume mobile phase B (µl)
1	4	96	4
2	6	94	6
3	8	92	8
4	11	89	11
5	13	87	13
6	17	83	17
7	20	80	20
8	23	77	23
9	27	73	27
10	31	69	31
11	36	64	36
12	40	60	40

Suppl. Table 4: Database search parameters used in Proteome Discoverer 2.2

Parameters	
Database	Rattus norvegicus (RefSeq V 108)
Search engines	Mascot and Sequest HT
Enzyme	Trypsin

Max. missed cleavage	2
Precursor mass tolerance (ppm)	10
Fragment mass tolerance (Da)	0.05
TMT10plex (Variable)	K
TMT10plex (Fixed)	Peptide N-termini
Oxidation (Variable)	M
Acetylation: +42.011 Da (Variable)	Protein N-Termini
Carbamidomethylation: +57.021 Da (Fixed)	C

Suppl. Table 5: Peptide mixture samples taken for pooling based on TMT label check

Sample ID	Normalization factor based on median abundance of TMT channels	Sample volume (µL)
G1	0.39	93
G2_1	0.15	120
G2_2	0.28	85
G2_3	0.20	118
G3_1	0.25	134
G3_2	0.18	105
G3_3	0.11	118
